# Investigating dynamical properties of the *Caenorhabditis elegans *connectome through full-network simulations

**DOI:** 10.1186/1471-2202-14-S1-P229

**Published:** 2013-07-08

**Authors:** James Kunert, Eli Shlizerman, J Nathan Kutz

**Affiliations:** 1 Physics, University of Washington, Seattle, WA 98195, USA; 2 Applied Mathematics, University of Washington, Seattle, WA 98195, USA

## 

The neuronal network of the nematode *Caenorhabditis elegans *(*C. elegans*) is comprised of 302 sensory, motor and inter-neurons. Near-complete connectivity data for the gap junctions and chemical synapses connecting these neurons (its connectome) have been resolved [1]. In addition, current experiments measure the response of various neurons to input stimuli. A description of these responses cannot be drawn from the static connectivity data alone. These studies suggest that computational modeling can assist in describing neural dynamics and their relation to the connectome. However, simulations of C. elegans neural dynamics are challenging since the single neuron dynamics do not appear to be characterized by standard spiking neuron models. Indeed, genomic sequencing and electro - physiological studies have consistently failed to observe classical Na^+ ^action potentials in C. elegans neurons [2].

**Figure 1:**
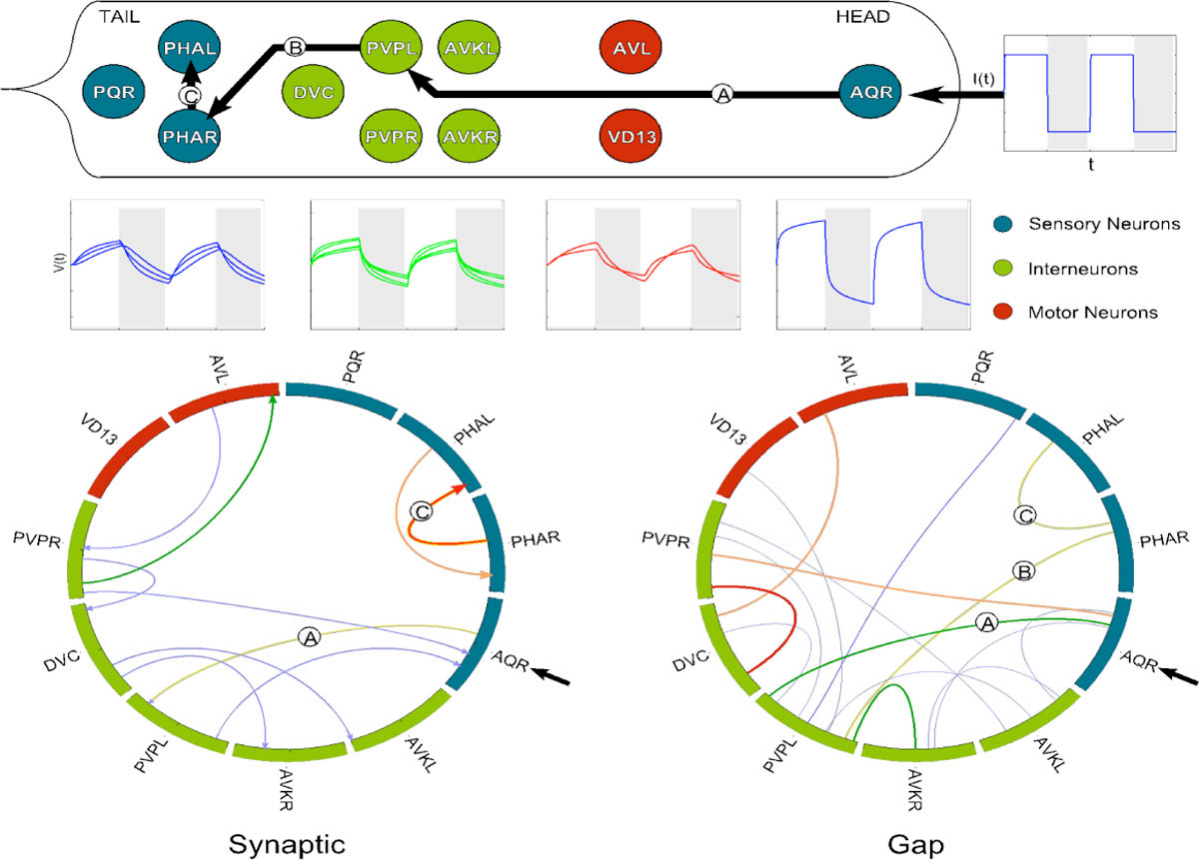
**Top**. Dynamic pathway from chemosensory neuron AQR positioned in the head to chemosensory neurons PHAL/R positioned in the tail. The AQR neuron is driven by periodic excitatory input and 11 neurons out of 302 that are *most responsive *to that input are shown. **Middle**. Voltage responses of the depicted neurons. **Bottom**. Circular plot of the synaptic and gap connectivity between the shown neurons. The blue, green, orange and red colors of the lines indicate a progressively higher number of connections. Connections contributing to pathway A→B→C are labeled as such. Note that AQR is not connected directly to any of the depicted tail sensory or motor neurons.

Our study combines the known connectome data [1] with a physiologically appropriate neuron model [3] to simulate the dynamics of the full neural network in response to stimuli over time. We model single neuron dynamics by graded electrical potentials using the findings of electrophysiological studies and biophysical considerations [3]. Since the parameters of the model are not well known, we first investigate their effect on the behavior and stability of the system. We use a genetic algorithm to explore this high dimensional parameter space. Once the parameters are set, we investigate the input-to-output response of the network. Specifically, we stimulate input sensory neurons, as is often done in experiments, and characterize the response elicited in the network. This is the first study of its kind *computationally *relating the sensory input with the resultant dynamical behavior of the inter- and motor-neurons. Figure 1 shows a prototypical example of the neural response when a chemosensory neuron AQR positioned in the head receives periodic input. A dominant pathway (A→B→C) shows how the signal propagates through inter-neurons to the tail chemosensory neurons PHAL/R. This example demonstrates that AQR and PHAL/R are highly correlated even with no direct static connection in the connectome. We call such a connection a "dynamical connection" between neurons, and our computational study discovers such dynamical connections.
